# Hydrothermal system dynamics at Pisciarelli fumarole field (Campi Flegrei): insights from geophysical and numerical modelling

**DOI:** 10.1038/s41598-026-46202-9

**Published:** 2026-04-03

**Authors:** Rosanna Salone, Antonio Troiano, Maria Giulia Di Giuseppe, Roberto Isaia, Rosa Di Maio

**Affiliations:** 1 Istituto Nazionale di Geofisica e Vulcanologia, Osservatorio Vesuviano, Naples, 80124 Italy; 2 Dipartimento di Scienze della Terra, dell’Ambiente e delle Risorse, Università degli Studi di Napoli Federico II, Naples, 80126 Italy

**Keywords:** Geophysical imaging, Thermo-fluid dynamic numerical simulations, Pisciarelli fumarolic-hydrothermal area, Campi Flegrei, Environmental sciences, Solid Earth sciences

## Abstract

The Pisciarelli fumarolic-hydrothermal area (Campi Flegrei caldera, Southern Italy), together with the adjacent Solfatara maar-diatreme, is a critical site due to the recent increase in volcanic activity. This includes very high CO_2_ fluxes (exceeding 600 tons/day), continuous morphological changes and frequent seismic events. This study aims to reconstruct the architecture and fluid dynamics of the Pisciarelli fumarole field by integrating geophysical imaging and thermo-fluid dynamic numerical modelling. A detailed 3D petrophysical model was developed from electrical resistivity, time-domain induced polarization and self-potential data, and was further constrained by existing volcanological and geochemical information. Thermodynamic simulations of a hot H_2_O-CO_2_ mixture circulating in the shallow hydrothermal system of Pisciarelli were carried out using the TOUGH2 numerical code with the EOS2 module. This modelling approach enabled detailed characterization of the system’s current dynamics, including temperature distribution, gas pressure, gas saturation levels, and fluid flow pathways. The results emphasize the pivotal role of the main fault system in controlling fluid accumulation and migration, which in turn drives the intense degassing activity observed at Pisciarelli. These findings provide new insights into the physical state of the Pisciarelli-Solfatara hydrothermal complex and contribute to assessing the potential for sudden explosive hydrothermal events under current conditions.

**Supplementary Information:**

The online version contains supplementary material available at 10.1038/s41598-026-46202-9.

## Introduction

**Fig. 1:**
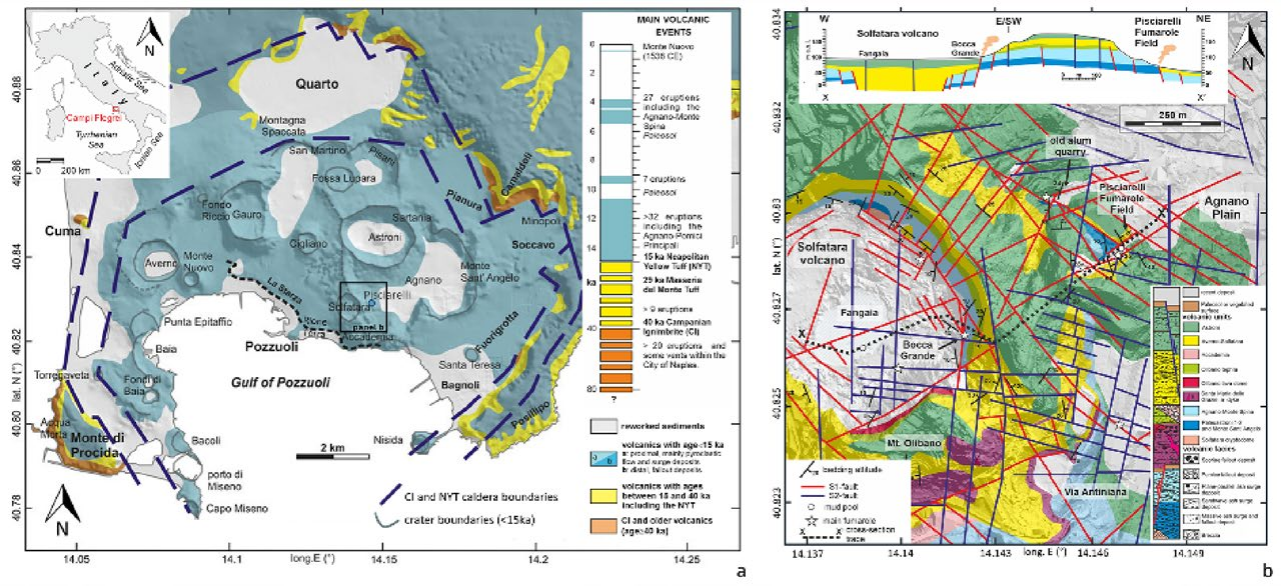
**a** Geological map of the Campi Flegrei caldera, showing the main volcano-tectonic structures and volcanic deposits. **b** Structural and geological details of the Solfatara-Pisciarelli-Agnano Plain sector, highlighting the main fault systems and fumaroles relevant to the current study. The figure was modified and adapted from^15^, with permission from the copyright holder.

The Campi Flegrei caldera (Southern Italy) is one of the most studied volcanic systems worldwide (Fig. 1). Its eruptive history includes the very large Campanian Ignimbrite eruption (∼40 ka;^1^, followed by a series of predominantly explosive events that contributed to the caldera morphology (e.g.^2^. These eruptive processes also led to the development of the main geothermal system located in the central sector of the caldera (e.g.^3,4^, which is considered the primary source of the current unrest phenomena^5^. The high population density and urbanization in the area, including parts of the cities of Naples and Pozzuoli as well as several smaller municipalities, significantly amplify the volcanic risk, which could be significant even in the case of minor events.

Ground deformation, surface degassing, and local seismicity have recurrently affected the area over the past several decades. Since 1982, pronounced ground uplift has been observed, reaching a maximum of 179 cm in the Rione Terra sector of Pozzuoli (Fig. 1a), accompanied by intense seismicity (more than 16,000 seismic events over a two-year span)^6–8^. This phase, which ended abruptly in 1984, was followed by a slow subsidence process lasting until 2005, when the ground level began to rise again, with the uplift rate increasing further in 2011^9^. This prolonged deformative phase, which is still ongoing, was not initially accompanied by an increase in seismicity, which remained relatively stable until 2022, when the occurrence of seismic events increased significantly, with ∼18,000 events from January 2023 to August 2025, including the most energetic event ever recorded in the caldera (Md 4.6, 30 June 2025)^10^. The observed deformative and seismic phenomena were accompanied by enhanced surface gas emissions, predominantly concentrated in the central sector of the caldera. Two primary zones were identified as the main sites of degassing: the Solfatara volcano and the Pisciarelli Fumarole Field (Fig. 1b)^11–13^. The Solfatara volcano, a maar-diatreme structure, exhibits relatively stable gas fluxes, accompanied by phenomena indicative of possible eastward fluid migration^14^. Consistently, the adjacent PFF, located to the east of Solfatara, has undergone progressive reactivation, emerging since 2011 as the principal site of surface degassing within the caldera. The main fumarole at PFF, known as the “Soffione”, currently emits over 600 tons/day of CO_2_, a flux comparable to that of a medium-sized arc volcano approaching eruptive conditions^12^. The area has also experienced abrupt morphological modifications, including the sudden opening of new fumaroles, rapid fluctuations in local spring water levels, and episodic mud emissions^15^. These phenomena have been accompanied by a significant increase in seismicity, which currently affects a large portion of the entire caldera. The convergence of these indicators, along with the high degree of urbanization, highlights the growing importance of the Pisciarelli area in the context of the current dynamics at Campi Flegrei, particularly in relation to the potential for hydrothermal/phreatic eruptive scenarios. Based on an analysis of typical phreatic eruptions, Valentine et al.^16^ estimated that, although explosions may occur over a range of depths, only those originating shallower than approximately 100 m are generally capable of ejecting material from the crater. At such depths, in areas such as the PFF, water may be in a thermodynamically favorable state to trigger explosive phenomena. Indeed, this state is compatible with sudden vaporization induced by even small changes in pressure, temperature, or permeability of the system, leading to transition from liquid to vapor and subsequent energy release.

In this framework, seismic activity plays a crucial role, as even small local earthquakes can induce structural changes that alter the geometry of the hydrothermal system. These perturbations may enhance the permeability of the complex fault network characterizing the area, thereby facilitating fluid redistribution and potentially triggering the abrupt release of pressurized fluids^17–19^. In addition, chemical alteration driven by the persistent circulation of reactive hot fluids exerts a significant influence on the system’s evolution. Over time, processes of mineral precipitation and dissolution dynamically modify the permeability of both faults and surrounding rock volumes in contrasting ways. While mineral precipitation tends to reduce permeability and reinforce fault sealing, dissolution processes or mechanically induced weakening can compromise structural integrity, promoting sudden permeability increases and substantial pressure redistribution^20,21^. Moreover, changes in heat flow due to variations in magmatic or hydrothermal activity can alter the thermal gradients within the system, thereby promoting localized vaporization in regions with abundant liquid water^22–24^. External factors, such as heavy rainfall or rapid changes in atmospheric pressure, can further destabilize the shallower portions of hydrothermal systems such as the PFF^25^. These processes, often acting in combination, may perturb the delicate equilibrium of the PFF, potentially leading to sudden phase transitions and the consequent release of thermal and mechanical energy.

Based on recent geophysical evidence, the present paper aims to develop a concise conceptual model of the PFF, through the construction of new thermo-fluid-dynamic models designed to investigate the underlying mechanisms and reconstruct the current state of the system. Since the advent of numerical simulators for multi-phase fluids in the 1980s, numerous studies have been carried out to characterize fluid circulation within volcanic edifices and to investigate features such as supercritical geothermal resources and steam-dominated zones, which are common in active geothermal systems^26^. These studies usually analyze the triggering of emissive and seismic phenomena driven by the ascent of magmatic fluids toward the surface. Although several studies have addressed the Campi Flegrei volcanic complex (e.g.^27–30^), including the Solfatara area, none has thoroughly examined the geological and fluid-dynamic complexities to the Pisciarelli Fumarole Field, one of the most active hydrothermal sectors of the caldera. Previous modelling efforts have not combined site-specific geophysical, geological, and geochemical constraints to resolve the structural heterogeneity and fluid-dynamic processes controlling present-day degassing at PFF, which is essential for understanding ongoing unrest and its potential hazard implications. This study addresses this gap by integrating multi-method geophysical observations with independent constraints to build a physically and geologically consistent model of the PFF hydrothermal system. In particular, we use numerical simulations to investigate the interaction between hydrothermal fluids and the complex geological and structural setting of the area, with particular attention to the existing intricate fault network. A 3D petrophysical model is constructed and used as input for simulations with the TOUGH2 numerical code, widely adopted for thermo-fluid dynamic modelling in geothermal and volcanic systems^31,32^. The current thermodynamic state of the PFF is reconstructed by setting physical constraints, such as the amount of fluid emissions recorded in the area and the temperatures measured at the ground surface. This approach allows for the estimation of the subsurface fluid distribution in terms of pressure, temperature, and vapor phase saturation, while shedding light on the role of the main fault system in fluid circulation. Such parameter estimations are crucial for improving our understanding of the Pisciarelli hydrothermal system and for identifying potential future variations that could serve as indicators of sudden system changes.

## Structural features of the PFF

The PFF lies along the eastern margin of the structural high that separates the Solfatara maar from the Agnano Plain. This area hosts a main fumarole, a mud pool, and a diffuse degassing zone, all located at the intersection between the NW-SE-trending Agnano caldera ring faults and the major ENE-WSW fault crossing the Solfatara crater (Fig. 1b). Since 2005, significant geochemical changes have been recorded at Pisciarelli, particularly a notable increase in CO_2_ flux^10^, which peaked at 600 tons/day^12^. Concurrently, the morphology of the degassing area has evolved, with a spatial enlargement involving both the fumarole and mud pool sectors, as well as the entire eastern flank of the Solfatara volcano. Seasonal fluctuations have also been observed, affecting the large crackling mud bubbles generated by the degassing phenomena. Furthermore, the Pisciarelli slope is affected by an active landslide system, responsible for generating several debris-flow deposits and mobilizing large boulders^15^.

**Fig. 2:**
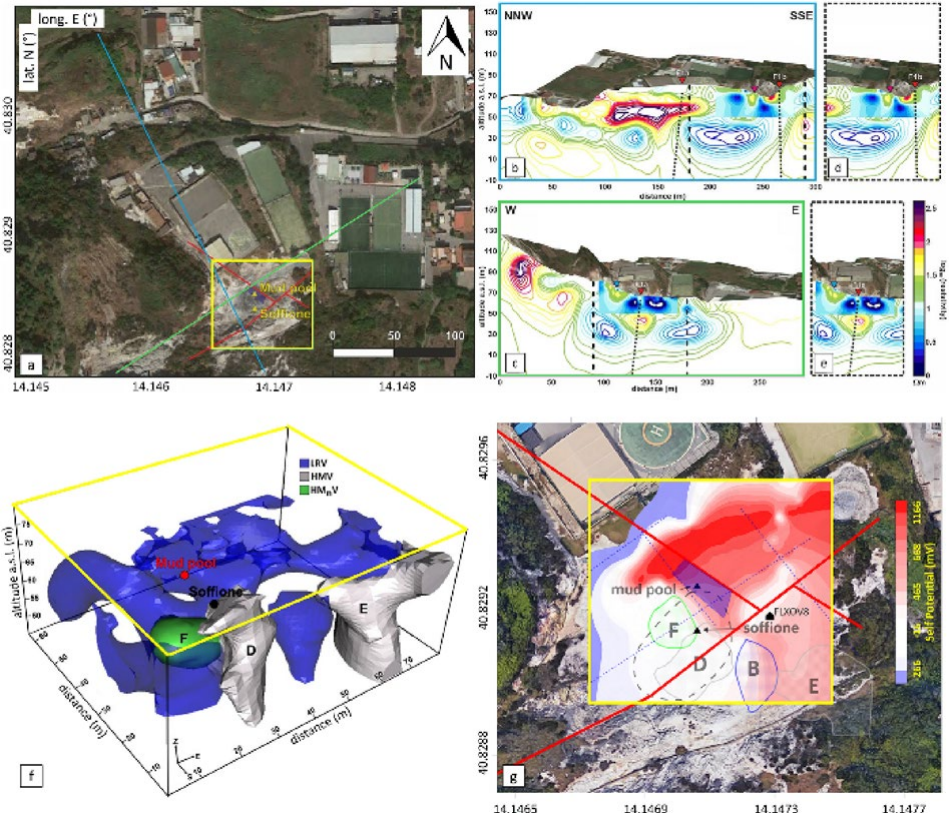
**a** Aerial view of Pisciarelli, showing the alignments of two of the three ERT profiles from^15^, reported as cyan and green solid lines. The main fault systems identified by^15^ are indicated with red solid lines. The yellow box delineates the area resolved by the high-resolution geoelectrical prospection presented in^37^. **b** and **c** 2D resistivity sections corresponding to the cyan and green transects shown in panel **a**, respectively. Black dotted squares indicate the areas magnified in panels **d** and **e**. Red triangles and black dotted lines mark the two fault systems. **f** 3D geoelectrical model of the Pisciarelli area showing the spatial distribution of key subsurface features, including low resistivity volumes (LRV; < 4 Ωm), high chargeability zones (HMV; bodies D and E, > 10 mV/V), and high normalized chargeability regions (HM_n_V; volume F, cut-off > 5). **g** SP anomaly map showing the traces of the main anomalies detected by the ERT and TDIP surveys (labeled with capital letters), along with the two main fault systems in the area. The figure was modified and adapted from^37^, licensed under a Creative Commons Attribution 4.0 International License.

In recent years, the number of studies focusing on the Pisciarelli sector has increased significantly^12,33–35^, with several geophysical investigations providing a detailed reconstruction of the PFF structure (Fig. 2a). In particular, Electrical Resistivity Tomography (ERT) surveys have significantly contributed to defining the volcano-tectonic setting and fluid circulation pathways. Isaia et al.^15^ identified a complex fault system in the first hundred meters of depth with NW-SE and NE-SW main directions, where damage zones act as preferential conduits for hydrothermal fluids, influencing the migration and accumulation of deep-seated gases and the emplacement of fumaroles and mud pools (Fig. 2b-e). In particular, the NE-SW-trending fault system was recognized as the boundary of an approximately 100–150 m wide resistive body (Fig. 2b) likely related to gas accumulation, as also suggested by larger-scale ERT data^36^. A subsequent high-resolution ERT survey, integrated with Time-Domain Induced Polarization (TDIP) tomography, self-potential (SP) and ground temperature mapping^37^, provided a 3D image of the shallow geothermal system down to ∼20 m. These investigations focused on the Soffione and mud pool areas (Fig. 2a), revealing a fluid migration channel connecting a deeper reservoir to the surface, overlaid by a clay cap formation that controls fluid circulation and gas emissions (Fig. 2f). Indeed, a prominent conductive structure, capped by a zone of high normalized chargeability (F in Fig. 2f) was identified and interpreted as a highly fractured channel along which shallow hydrothermal fluids form a water/gas plume beneath the clay cap that modulates degassing at the Soffione. In addition, the surveys revealed a relatively resistive overburden extending 2–3 m below the surface, overlying a more conductive layer with elongated anomalies aligned with the two main fault systems described by^15^. The SP map (Fig. 2g) revealed a marked peak-to-peak signal excursion, with the NE and SW sectors showing a positive and negative trends, respectively. This pattern correlates well with fault traces mapped through geological observations, suggesting that the SP anomalous behaviour reflects the influence of a main fault system. The NE sector was interpreted as a purely hydrogeological zone, while the SW sector was attributed to the main upflow zone for fluids ascending from a deeper reservoir. The identified fault system, therefore, would act as a semi-permeable boundary, exerting a *barrier effect* by partially impeding the northward migration of fluids and promoting their accumulation within the reservoir^15,33,37^.

## Materials and methods

### Thermo-fluid dynamic numerical modeling

A naturally fractured geothermal reservoir can be modelled as a series of interconnected porous blocks separated by fractures, through which fluids circulate under pressure gradients and heat is transferred by conduction and convection. Heat flow entering a rock-fluid volume is partitioned so that thermal equilibrium between rock and fluid is approximately maintained. In this framework, the rock matrix acts as both a fluid reservoir, feeding the fractures via pressure-driven exchange, and a heat reservoir, transferring thermal energy to the circulating fluids.

The state and evolution of similar systems have often been modelled using physical-mathematical approaches that solve coupled multi-phase, multi-component fluid flow and heat transport problems using appropriate numerical codes. Among these, TOUGH2 (Transport Of Unsaturated Groundwater and Heat) is one of the most widely used. This code computes mass and heat exchange associated with multi-dimensional flows of two-phase (gas and liquid) mixtures of multiple components in a porous medium with assigned permeability^38,39^. TOUGH2 assumes local thermodynamic equilibrium between fluid and rock matrix and employs the *integral finite-difference method* to discretize mass and energy balance equations. The solution provides the primary thermodynamic variables (e.g. pressure, temperature, CO_2_ partial pressure) as functions of time at the centres of the elementary cells that discretize the physical domain. Geometrical properties required to define the flow system (cell volumes, interface areas, intercellular distances, and gravity components along the connection) are specified by the user. A comprehensive description of the fundamental governing equations solved by TOUGH2 for multi-component, multi-phase fluid flow and their coupling with heat transport is given in^38^. Fluid properties enter these equations exclusively through physical parameters (density, viscosity, and enthalpy), computed as functions of the thermodynamic conditions – typically pressure and temperature – according to the steam-table formulations^40^.

**Fig. 3:**
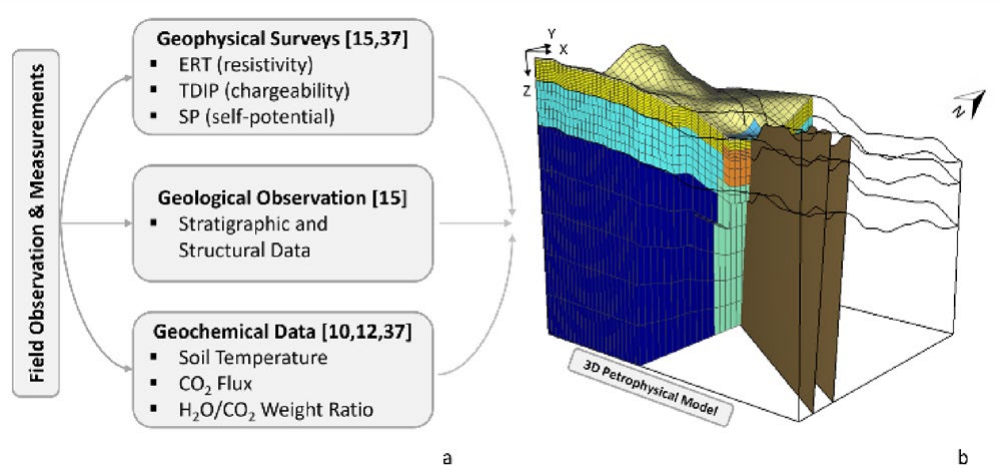
Flowchart of the numerical simulation workflow. **a** Initial constraints derived from the integration of electrical resistivity tomography (ERT), time-domain induced polarization (TDIP) and self-potential (SP) data, used to define a consistent physical model of the hydrothermal system and to identify anomalous zones associated with fluid pathways. **b** Resulting 3D petrophysical model, obtained by combining the geophysical dataset with stratigraphic, structural and geochemical information from previous studies and used as the basis for the thermo-fluid dynamic simulations.

In this paper, we apply this approach to assess the current thermodynamic state of the shallower PFF geothermal system, constrained by geophysical, geological and geochemical data (Fig. 3). The petrophysical parameters of the main PFF structures (Table 1), as well as the initial and boundary conditions, were derived from previous studies (e.g. ^12,27,30,41–46^), which also allowed a simplified representation of the system geometries. Fluid circulation is modelled as hot magmatic fluids entering the base of a permeable channel from the deeper geothermal system. As discussed in the Permeability architecture, parameterization, and sensitivity section, flow is primarily controlled by the spatial distribution of rock permeability, leading to focused discharge at the Soffione vent and minor diffuse degassing in the surrounding area.

**Fig. 4:**
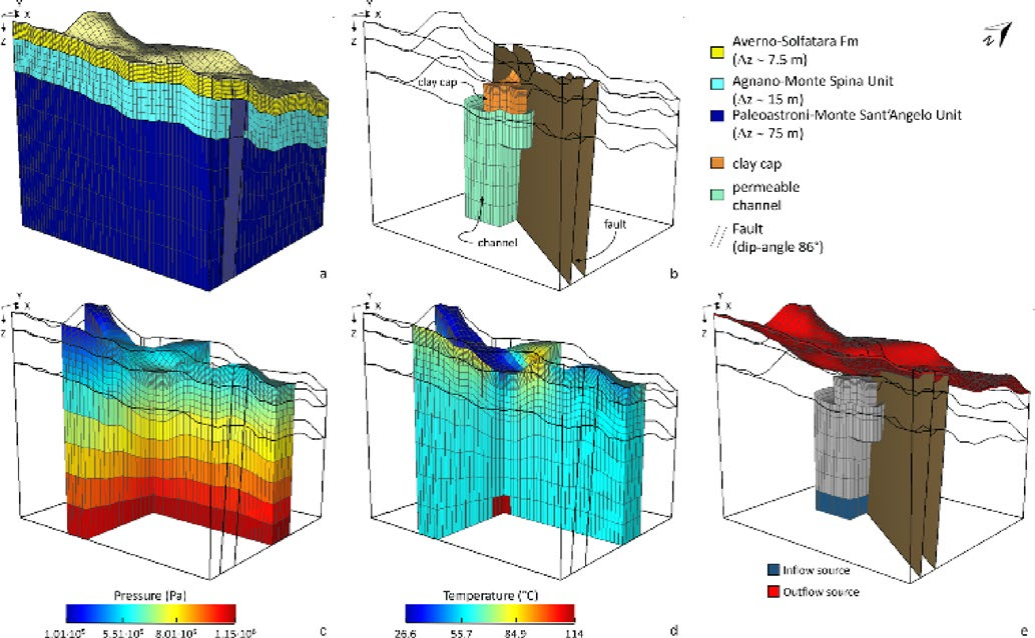
**a** 3D petrophysical model used to simulate the ascent of a hot CO_2_-rich gas mixture within the Pisciarelli hydrothermal system. **b** 3D view of the internal structure of the model, showing: (i) the highly fractured zone that allows shallow fluids to form a water/gas plume (i.e. the channel, shown in light green); (ii) the clay cap overlying the fluid upwelling channel (in orange); and (iii) the main fault system that governs fluid migration and acts as a permeability barrier (represented by slice planes). **c** Hydrostatic pressure gradient and **d** temperature distribution adopted as initial conditions. **e** Assumed source geometry for the influx of a hot H_2_O-CO_2_ mixture (blue cells). Red cells indicate locations where a mass flux emission rate is imposed to ensure physically consistent boundary conditions.

As shown in Fig. 4, the Pisciarelli area was represented as a prismatic volume of ∼110 × 90 × 100 m^3^. These dimensions slightly exceed those of the geophysical surveys in^37^, to incorporate additional features revealed by deeper geoelectrical investigations^15^. The volume was discretized into a regular grid comprising 33,000 elementary cells, each serving as a control volume for the integral finite-difference formulation implemented in TOUGH2 numerical code. The computational domain was defined in a Cartesian coordinate system with the origin located at the SW corner of the model, the X-axis oriented W-E, the Y-axis in the S-N direction, and the Z-axis oriented downward (Fig. 4a). The grid consisted of 55 cells of 2 m along the X-direction, 30 cells of 3 m along the Y-direction, and 20 layers along the Z-direction with thicknesses ranging from 0.75 m to 15 m. To ensure numerical stability and convergence, the computational domain, which includes the real topography of the area, was laterally extended with larger buffer cells at the boundaries.

This discretization represents a reasonable compromise between computational efficiency and the need to resolve the main geological and structural features controlling fluid circulation, including the fault zone, the shallow low-permeability cap, and the major permeability contrasts inferred from geophysical data^15,37^. The choice of a regular, structured grid ensures numerical stability and transparency in solving the governing mass and energy balance equations while remaining consistent with the scale and resolution of available observational constraints.

The stratigraphic configuration implemented in the model reflects the main lithostratigraphic units of the study area, as described in^15^ and shown in Fig. 4a. The sequence comprises: (i) the Averno-Solfatara Formation, ∼7.5 m thick; (ii) the Agnano-Monte Spina Unit, with a thickness of ∼15 m; and (iii) the Paleoastroni-Monte Sant’Angelo Unit, representing the basement.

The upflow channel was modelled as a 22 × 33 × 81 m^3^ prismatic body that widens upward (i.e. 36 m along X-direction) beneath the Soffione-mud pool sector (Fig. 4b). A clay-rich cap overlaps the channel as a laterally continuous, shallow layer (shown in orange in Fig. 4b) measuring 16 × 21 × 13 m^3^. Consistent with the structural framework described in the Structural features of the PFF, the numerical model explicitly incorporates the NW-SE-trending fault system recognized as the main structural control on hydrothermal fluid migration, accumulation and surface degassing at the PFF. Accordingly, the fault zone was considered as a homogeneous, isotropic volume bounded by two parallel NW-SE planes with a dip angle of 86° (Fig. 4b), as inferred from geological and structural analyses^15^. The permeability values assigned to the different structural elements, including the fault zone, are described below.

We simulated two-component (H_2_O-CO_2_) and two-phase flow. Pressure, temperature, and CO_2_ partial pressure were used as primary variables. Initial conditions (Fig. 4c, d) comprised a hydrostatic pressure gradient and a surface-constrained temperature field based on measurements in^37^, applied as the top boundary conditions. To account for local thermal anomalies, a temperature of 114 °C was assigned exclusively to cells corresponding to fluid source locations^30^. The initial CO_2_ partial pressure was set uniformly to 35 Pa, consistent with atmospheric equilibrium and previous modelling studies^27,30^. A preliminary steady-state run confirmed a realistic geothermal gradient (∼0.105 °C/m;^30^) and a physically consistent CO_2_ distribution within the domain^10,11,13^.

To reproduce deep injection and surface discharge, pressure variations were restricted to two regions: (i) the base of the source channel, and (ii) the Soffione-mud pool sector and its surroundings, where surface gas emissions are most intense (Fig. 4e). A constant mass flow rate was injected at the base of the channel (blue cells in Fig. 4e), while a mass flux condition was imposed on the upper boundary cells (red cells in Fig. 4e) to represent surface discharge. Elsewhere, the lateral and basal boundaries were treated as insulated and impermeable. To minimize the impact of boundary artefacts, we added large volume cells at the outermost lateral and top grid blocks, excluding the outflow cells. The total amount of fluids injected into and discharged from the system is discussed below and summarized in Table 2.

### Numerical simulations

We performed simulations with TOUGH2 in a three-dimensional computational domain, using the conceptual model and the initial and boundary conditions described above. The aim was to replicate the ascent of a hot H_2_O-CO_2_ mixture and quantify the associated heat and mass transport for a data-constrained injection. Runs proceeded to a quasi-steady state, taken as a proxy for the current thermodynamic state. The injected mass and simulation time were constrained by the physics of the system and by the monitoring data used as inputs and validation targets.

Emissions were constrained using INGV surveillance data^10^, adopting a CO_2_ flux of ∼600 tons/day^12^ and H_2_O/CO_2_ mass ratio of 1.6. Although this ratio can vary over time, the value of 1.6 was selected as a baseline, consistent with previous numerical simulations performed in the Campi Flegrei area^29,30,47^, as well as with the fumarolic gas composition measured at Solfatara during the reference period^48^. The adopted CO_2_ flux reflects the order of magnitude of the current degassing regime at Pisciarelli and was used to reconstruct the thermodynamic state of the system at the time of the geophysical surveys (2020), assuming quasi-steady conditions rather than explicitly modelling its temporal evolution.

Accordingly, we enforced a quasi-steady mass balance, whereby deep inflow matches surface discharge, which is concentrated at both the Soffione vent and mud pool area with a minor diffuse degassing in the surrounding sectors. Guided by CO_2_ flux monitoring data^10^, we imposed a continuous injection of 9.68 kg/s of a 130 °C H_2_O-CO_2_ mixture into source cells at ∼100 m depth at the channel base (Fig. 4e), with specific enthalpies of 4.79·10^7^ J/kg for H_2_O and 1.19·10^7^ J/kg for CO_2_. The corresponding injection and discharge conditions adopted in the numerical simulations are summarized in Table 2.

### Permeability architecture, parameterization, and sensitivity

At the Pisciarelli Fumarole Field (PFF), permeability is the primary factor controlling fluid circulation and heat-mass transfer. In the model, we assigned permeability at two levels: (i) lithostratigraphic units and (ii) structure-specific bodies (i.e. upflow channel, clay cap, and main fault), as defined by the integrated ERT and TDIP interpretation from^37^ (Fig. 2f), and the mapped fault framework (Fig. 1b)^15,37^. Literature-derived background permeabilities were assigned to the lithostratigraphic units (Table 1)^42,44–46^. The resulting conceptual model is encoded by structure-specific values: the upflow channel is the most permeable element (1 × 10^− 13^ m^2^), focusing the ascent of a H_2_O-CO_2_ mixture; the clay cap has lower permeability (5 × 10^− 14^ m^2^), acting as a partially permeable seal that modulates surface degassing; and the main fault has reduced permeability (1 × 10^− 17^ m^2^), reproducing its present barrier-like behaviour, as inferred from SP and resistivity contrasts^15,37^. Other petrophysical properties (i.e. porosity, density, heat capacity and thermal conductivity) were kept fixed and followed representative literature values (Table 1).

To assess the robustness of the modelling results, we conducted a series of sensitivity tests by systematically varying the key controlling parameters. In particular, clay cap permeability was varied over approximately one order of magnitude (10^− 16^ – 10^− 17^ m^2^), while alternative fault hydraulic configurations were explored, spanning permeable to low-permeability end-members (10^− 12^ – 10^− 17^ m^2^), consistent with literature values and geophysical interpretations. For each configuration, the deep H_2_O-CO_2_ injection flux was adjusted accordingly to maintain degassing regimes compatible with the observed surface CO_2_ emissions. Step-by-step variations of these parameters result in gradual and physically consistent changes in pressure build-up beneath the cap, plume geometry, and surface degassing intensity, without altering the first-order architecture of fluid migration inferred from the reference model. Acceptable configurations were those that remained numerically stable and reproduced both the observed CO_2_ flux at the surface and its spatial maximum at the Soffione (Fig. 2g). Although absolute pressure values and flux magnitudes vary across the tested scenarios, the main conclusions regarding the upflow channel role, the partially permeable clay cap, and the barrier-like behaviour of the fault system remain robust under reasonable parameter variations. Values of all parameters used for the sensitivity tests and the corresponding quantitative results are reported in the Supplementary Information (Tables S1–S2; Figs. S1–S2).

**Table 1:** Parameters characterizing the discretized model shown in Figs. 4a-b.

Layer	Density (kg/m^3^)	Porosity	Permeability (m^2^)	Wet Heat Conductivity (W/m°C)	Specific Heat (J/kg°C)
Averno-Solfatara	1600.0 [45]	0.45^45^	1.00·10^− 14^^45^	1.15^45^	900^45^
Agnano-Monte Spina	2500.0 [46]	0.785^46^	1.00·10^− 15^^46^	1.15^45^	900^45^
Paleoastroni-Monte Sant’Angelo	2400.0 [46]	0.485^46^	1.00·10^− 16^^46^	1.15^45^	900^45^
Structure					
Clay cap	1600.0 [44]	0.42^41^	5.00·10^− 14^^42^	1.28^44^	860^43^
Channel	2500.0 [46]	0.785^46^	1.00·10^− 13^	1.15^45^	900^45^
Fault	2400.0 [46]	0.485^46^	1.00·10^− 17^	1.15^45^	900^45^

**Table 2:** Parameters characterizing the mass fluxes imposed at the injection and discharge source cells shown in Fig. 4e.

Source Location	Process	*N*. cells	Total H_2_O+CO_2_ flux (kg/s)	Total H_2_O flux (kg/s)	Total CO_2_ flux (kg/s)
Channel base	Injection	121	9.68	6.05*	3.63*
Soffione + mud pool	Discharge	29	6.09	–	–
Surrounding area	Discharge	1,621	3.24	–	–

*for each injection and discharge source, the H_2_O and CO_2_ mass flux assigned to an individual grid cell is calculated by dividing the total mass flux reported in the table by the number of source cells.

## Results and discussion

**Fig. 5:**
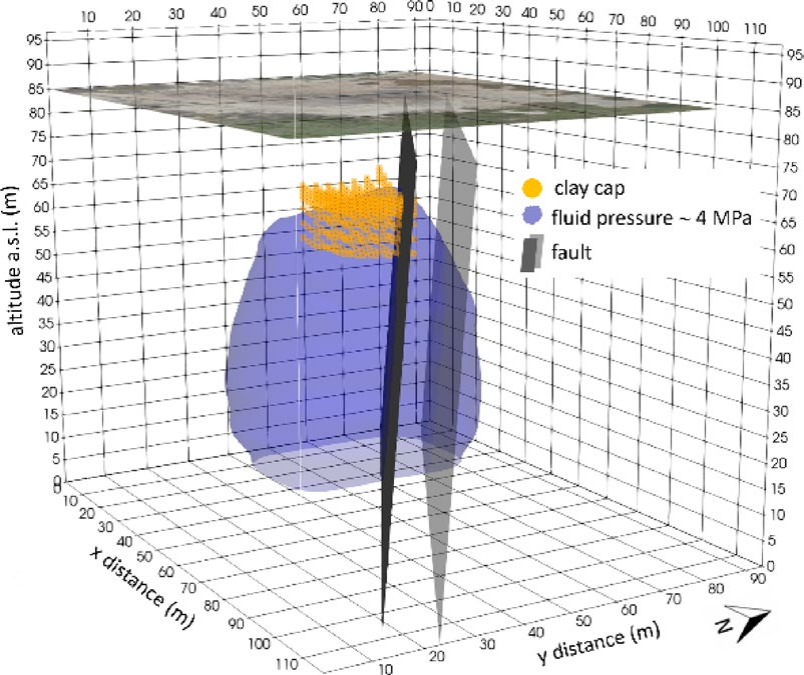
Fluid pressure distribution at the final state of the numerical simulation. The blue isosurface delineates a fluid-filled volume with a pressure of at least 4 MPa. Orange dots mark the position of the clay cap, while the two gray planes represent the fault system zone, modeled as a low-permeability volume. The figure was created using the ParaView v6.1 software (https://www.paraview.org).

Figure 5 shows the fluid pressure distribution obtained at the final state of the numerical simulation. The blue isosurface encloses a pressurized volume with P ≥ 4 MPa, which corresponds to the estimated pressure for the Campi Flegrei hydrothermal system (i.e. ∼44 bar;^12^). Its geometry is closely constrained by the structural features of the system, the clay cap (orange dots) and the fault zone (gray planes). At depth, the surface becomes gently domed, defining a broad accumulation zone near the channel base and indicating overpressure beneath the cap. This pattern is consistent with a partially permeable cap that limits vertical escape while permitting limited leakage towards the surface. The result is further supported by the sensitivity analysis in the Supplementary Information, which suggests that Pisciarelli clay cap should be regarded as a partially permeable barrier rather than a fully impermeable seal. This would allow limited fluid leakage towards the surface under the present conditions.

**Fig. 6:**
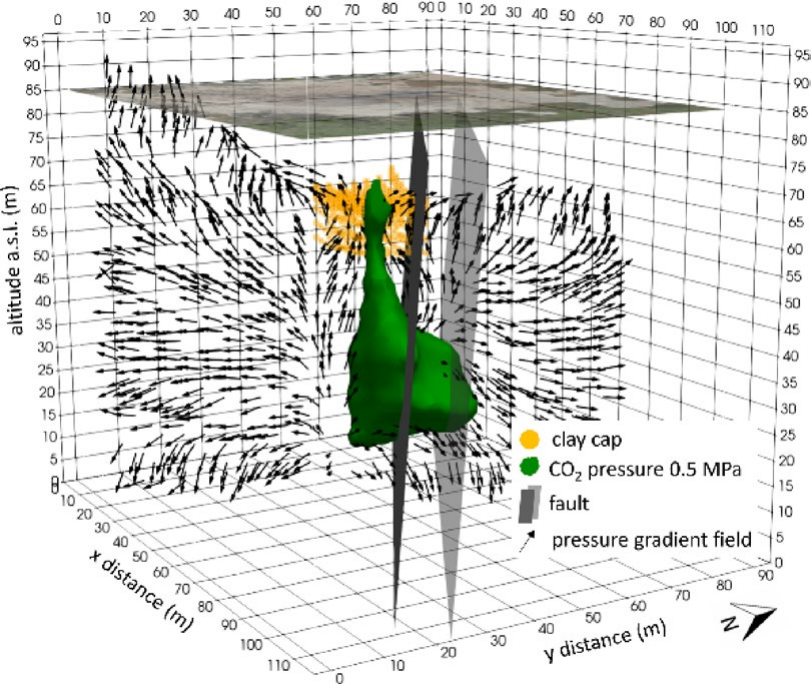
CO_2_ partial pressure distribution at the final state of the numerical simulation. The green isosurface corresponds to the 0.5 MPa pressure level. Orange dots indicate the location of the clay cap, while the two gray planes represent the fault zone, modeled as a low-permeability volume. Black arrows illustrate the pressure gradient field on a plane orthogonal to the fault system. The figure was created using the ParaView v6.1 software (https://www.paraview.org).

Figure 6 shows the distribution of CO_2_ partial pressure at P = 0.5 MPa (green isosurface). A broad accumulation forms at the base of the channel, where pressure and permeability conditions favour CO_2_ build-up; upward, the plume narrows and displays a clear lateral offset beneath the clay cap, departing from the channel’s central axis.

The pressure gradient field (∇P), shown on a cross-section orthogonal to the fault plane (black arrows), explains this geometry. In the deeper part of the channel (10–40 m a.s.l.), vectors are mainly vertical, promoting vertical gas ascent. In contrast, in the uppermost part, they converge towards the centre of the cap, suggesting lateral focusing immediately beneath the caprock. At the outer edges of the channel, the vectors rotate to a predominantly horizontal orientation, delineating lateral pathways. As a result, the plume follows the channel edge rather than its median axis. This pattern is consistent with the reorientation of the pressure field induced by the partially permeable clay cap, which promotes overpressure and lateral redistribution, and by the neighbouring fault zone, which dampens vertical gradients across its core. At the channel boundary, the steeper local vertical component of ∇P provides a more direct and energetically favourable upward path, whereas the inward-converging gradients within the central channel generate zones of higher confinement, reducing ascent efficiency and promoting lateral deflection. Overall, the resulting plume geometry reflects the combined effect of buoyancy, pressure-gradient organization and structural heterogeneity (channel-cap-fault coupling) on CO_2_ accumulation and release dynamics.

**Fig. 7:**
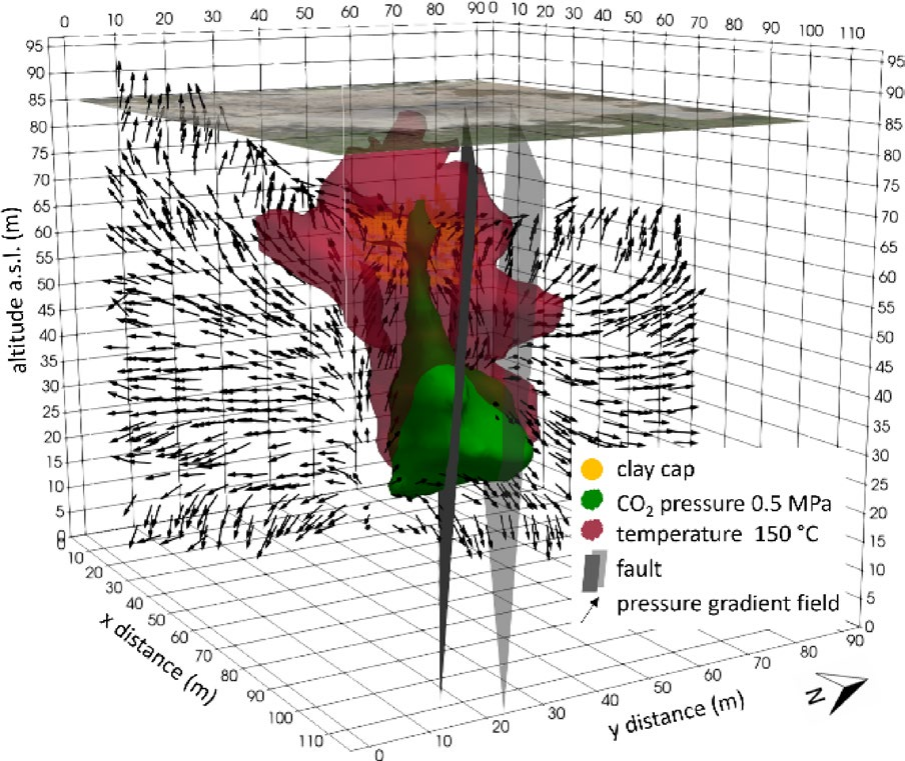
Temperature distribution corresponding to the final state of the numerical simulation. The 150 °C isosurface is shown in red, while the 0.5 MPa CO_2_ partial pressure isosurface is shown in green. Orange dots indicate the location of the clay cap. The two gray planes define the fault system zone, modeled as a reduced-permeability volume. Black arrows represent the pressure gradient field on a plane orthogonal to the fault system. The figure was created using the ParaView v6.1 software (https://www.paraview.org).

Figure 7 displays the T = 150 °C isosurface (red) together with the P_CO2_ = 0.5 MPa plume (green). These thresholds were selected based on the simulation results: 0.5 MPa corresponds to the maximum CO_2_ overpressure attained at the end of the modelling, while 150 °C coincides with the highest temperature reached within the system. Although these values correspond to maxima, they are not confined to localized peaks but instead characterize the pressurized and heated core of the entire upflow channel, which evolves toward a relatively homogeneous state of overpressure and thermal perturbation under the imposed steady conditions. While the two fields are spatially associated, they are not coincident: the 150 °C surface envelops the CO_2_ plume and extends further laterally, forming a shallower lobe immediately beneath the clay cap. This reflects their distinct transport behaviours and the influence of the permeability architecture.

Guided by permeability contrasts and buoyancy/pressure gradients, CO_2_ rises within the upflow channel and tends to be retained beneath the cap. Here, the pressure field is reoriented by cap-induced overpressure and the neighbouring fault (see Figs. 5 and 6). This produces a laterally offset plume and a deep accumulation zone at the base of the channel. Conversely, heat is transferred via convection within the fluids and then conducted into the surrounding rock. As a result, the thermal anomaly extends beyond the gas conduit and approaches closer to the cap than the CO_2_ isosurface.

The resulting distribution suggests that the cap’s reduced permeability causes CO_2_ and pressure to build up beneath it, while the rock matrix redistributes heat laterally and vertically from the upflow zone. Therefore, areas of high temperature do not necessarily coincide with the highest CO_2_ partial pressure near the cap because thermal diffusion can outpace the ascent of gas.

**Fig. 8:**
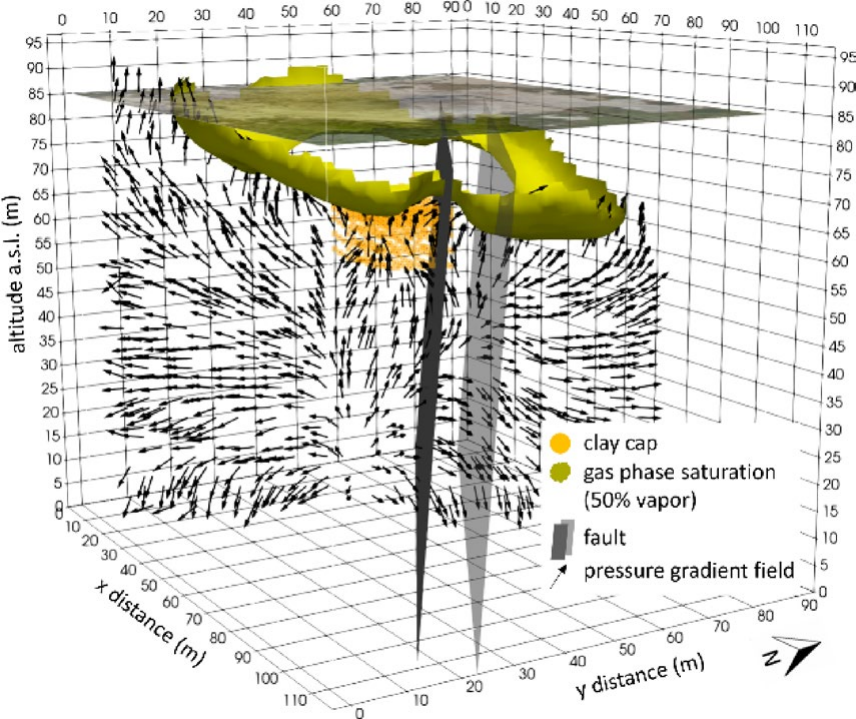
Gas phase saturation at the final state of the numerical simulation. The yellow isosurface marks a region where both liquid and gaseous phases coexist, with a vapor phase of at least 50%. Orange dots indicate the position of the clay cap, while the two gray planes delineate the fault system zone, modeled as a reduced-permeability volume. Black arrows represent the pressure gradient field on a plane orthogonal to the fault system. The figure was created using the ParaView v6.1 software (https://www.paraview.org).

Figure 8 shows the distribution of gas phase saturation (SG). The yellow isosurface marks a region where liquid and gaseous phases coexist, with a vapor phase of at least 50%. Within the channel, high pressures maintain liquid-dominated conditions, whereas immediately beneath the clay cap, fluids spread laterally beyond the channel margins. In this peripheral zone, the pressure drop favours vapour enrichment, forming a ring-shaped, two-phase volume around the channel. This pattern is consistent with lateral redistribution beneath the cap and the influence of the fault corridor.

**Fig. 9:**
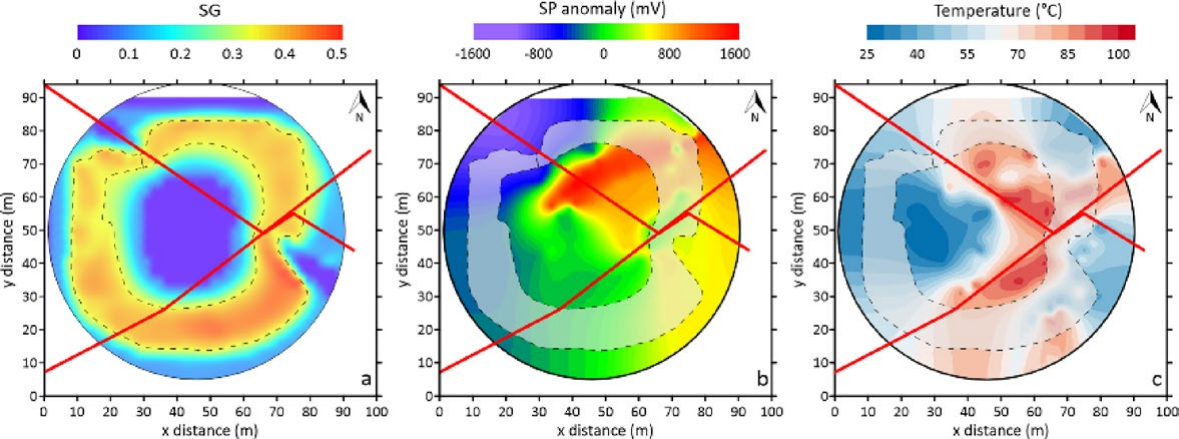
**a** 2D projection of the region with gas phase saturation (SG) with a vapor phase of at least 50% (yellow isosurface in Fig. 8) superimposed on **b** the SP anomaly map and **c** the soil temperature distribution of the PFF, both redrawn from^37^. The overlaid red lines indicate the main fault systems as reported by^15^.

Figure 9 compares the plan-view footprint of the high gas-saturation zone (dashed lines in Fig. 9a), reconstructed by the numerical simulation, with independent SP (Fig, 9b) and soil-temperature (Fig. 9c) anomaly maps redrawn from^37^. The SP contours resemble the spatial distribution of the gas saturation zone derived from the model (Figs. 9a, b), particularly in the ring-shaped structure surrounding the central fluid-dominated zone. This spatial correspondence suggests that increased gas saturation significantly contributes to the observed SP anomalies. However, localized deviations are observed on the western side of the fault, where the match between SP anomalies and modelled gas saturation is weaker. These discrepancies likely reflect spatial variability in subsurface fluid pathways and permeability, which is further discussed below in the context of fault-sealing processes. The comparison with the soil-temperature map (Fig. 9c) further strengthens the reliability of the numerical model, as areas of higher simulated gas saturation align with zones of elevated temperatures, particularly along the fault systems, highlighting the previously discussed coupling between gas transport and heat transfer mechanisms. Overall, the agreement between observations and simulations provides independent support for the model’s ability to reproduce subsurface gas migration and accumulation processes within the Pisciarelli hydrothermal system.

The pressure gradient vectors (Figs. 6, 7 and 8) elucidate the structural control exerted by the fault system. When modeled as a reduced-permeability corridor, the fault suppresses vertical pressure gradients across its core and promotes lateral redirection of flow along the channel-fault interface, which is more pronounced on the SW side, adjacent to the permeable channel. Conversely, on the NE side of the channel, pressure gradients remain predominantly horizontal and buoyancy-driven ascent dominates, with limited structural interference. This pressure-field organization favours CO_2_ accumulation and overpressure near the fault interface, imparting a lateral deflection to the rising plume. At shallow depth, interaction with the clay cap further enhances lateral redistribution, reinforcing the fault’s role in modulating both vertical and horizontal pressure gradients and, ultimately, the architecture of fluid pathways.

These model-derived flow patterns provide a physical basis for interpreting the different fluid flow regimes observed in the two sectors of the Pisciarelli Fumarole Field (PFF), as highlighted by geophysical investigations^15,37^, which may be attributed to the fault acting as a zone of reduced permeability. In general, faults can behave as conduits, barriers, or combined conduit-barrier systems^17^, depending on the structural properties of the fault core, where most of the displacement is accommodated, and the surrounding damage zone. When fractures within the fault core and damage zone are sufficiently open and interconnected, the fault may act as an open conduit, enabling efficient upward fluid transport. Transient processes such as intense or sustained seismicity can further facilitate this behaviour by widening existing fractures or generating new ones, thereby increasing permeability. However, continued ascent of hot, mineral-rich fluids can progressively modify this initial high-permeability state. Mineral precipitation within fractures and pore space promotes sealing of the fault core, reducing permeability over time and favouring a transition toward barrier-like behaviour. Within this combined conduit-barrier framework, the fault may initially channel fluids upward while progressively inhibiting cross-fault flow as sealing advances. The simulated thermo-fluid dynamic circulation patterns (Figs. 6, 7 and 8) are consistent with this evolutionary scenario, indicating that the fault at Pisciarelli currently behaves predominantly as a permeability barrier. This suggests that ongoing sealing processes exert a primary control on the present-day permeability architecture and fluid circulation within the fault zone.

Taken together, the barrier-like fault, cap-induced overpressure, and the coexistence of liquid- and vapour-dominated zones define a delicate dynamic equilibrium. In this configuration, even modest perturbations in pressure, permeability, or temperature may alter phase relations and redistribute stored energy by reorganizing flow paths and overpressure domains, highlighting the intrinsically dynamic and potentially unstable nature of the shallow hydrothermal system at PFF.

From a monitoring perspective, our results indicate where observational efforts should be focused, and which variables provide the most diagnostic information. The Soffione sector and the channel-fault interface are identified as priority areas, as the model identifies them as zones of maximum pressure build-up and the most focused CO_2_ ascent. Immediately above the clay cap, the ring-shaped two-phase zone is expected to expand or contract in response to variations in pressure and permeability, making it a sensitive indicator of evolving subsurface conditions. In parallel, the fault corridor organizes lateral flow and can therefore modulate the surface expression of degassing. An effective surveillance strategy involves combining continuous measurements of CO_2_ flux and soil temperature with repeated SP, ERT and TDIP surveys focused on the channel-fault interface and the shallow cap domain. Interpreted jointly with the modelled P-T-SG fields, these observations would allow early recognition of shifts in overpressure pockets or two-phase conditions. Integrating these data streams into a centralized framework with near-real-time updates would further enhance the identification of precursory changes and support iterative model refinement, ensuring that forecasts remain consistent with the evolving subsurface dynamics.

## Conclusions

The Pisciarelli area, located within the Campi Flegrei caldera (Southern Italy), has undergone marked changes over the past two decades and is widely regarded as a potential site for the opening of new vents and for hydrothermal and/or phreatic explosions. In this context, quantifying the current thermodynamic state of the shallow system and assessing the structural controls on fluid pathways are crucial steps toward refining conceptual models and improving hazard assessment.

The thermo-fluid dynamic simulations presented in this work, based on a 3D petrophysical model and constrained by volcanological, geophysical, and geochemical observations, provide a quantitative snapshot of the actively degassing Pisciarelli Fumarole Field. Beyond reconstructing the structural architecture (i.e. channel, clay cap and fault zone), the integrated approach resolves the distribution of pressure, CO_2_ partial pressure, temperature, and gas saturation at shallow depth, thereby illuminating the dynamics of fluid ascent, trapping, and lateral redistribution under present conditions.

In this setting, four main features characterize the shallow configuration: (i) a contiguous pressurized volume (P ≥ 4 MPa) beneath the clay cap, with a gently domed geometry centered near the channel base; (ii) a CO_2_-rich plume (P_CO2_ = 0.5 MPa) ascending within the upflow channel and laterally offset beneath the cap; (iii) a ring-shaped two-phase zone (SG ≥ 0.5) immediately above the cap, where pressure decrease favours vapour enrichment; and (iv) a first-order structural control exerted by the fault zone, which limits northward propagation of overpressure and re-orients flow along the channel-fault interface.

Together, these features support an updated conceptual model in which a partially permeable cap concentrates pressure and CO_2_ below it, while a barrier-dominated fault controls lateral redistribution at shallow depth. The model outputs provide physics-based inputs for forward scenario modelling. Specifically, transient simulations exploring (a) pressure perturbations at depth, (b) variations in cap or fault permeability (e.g., fracture opening vs. sealing), and (c) thermal transients can quantify overpressure redistribution, the migration of two-phase conditions, and changes in surface degassing. Such analyses would support impact forecasting for hydrothermal and/or phreatic events by identifying where energy is stored and how it may be released under plausible perturbations, thereby improving estimates of event magnitude and potentially affected areas.

## Supplementary Information

Below is the link to the electronic supplementary material.

Supplementary Material 1

## Data Availability

All the parameters required to reproduce the numerical simulations are reported in the manuscript and Supplementary Information. Additional details on the model implementation can be provided by the corresponding author upon request.
