# Supplementary material for: Hydrothermal system dynamics at Pisciarelli fumarole field (Campi Flegrei): insights from geophysical and numerical modelling
Source: Sci Rep. 2026 Apr 3;16:15852. doi: 10.1038/s41598-026-46202-9 (PMC13195099; doi:10.1038/s41598-026-46202-9)
Supplement: Supplementary file 1 — Supplementary Material 1 [file 41598_2026_46202_MOESM1_ESM.docx]

**Supplementary** **Information**

**Hydrothermal System Dynamics at Pisciarelli Fumarole Field (Campi Flegrei): Insights from Geophysical and Numerical Modelling**

**R. Salone^1,2*^, A. Troiano^1^, M. G. Di Giuseppe^1^, R. Isaia^1^, and R. Di Maio^2^**

^1^Istituto Nazionale di Geofisica e Vulcanologia, Osservatorio Vesuviano, Naples 80124, Italy.

^2^Dipartimento di Scienze della Terra, dell’Ambiente e delle Risorse, Università degli Studi di Napoli Federico II, Naples 80126, Italy.

*Corresponding Author: R. Salone ([rosanna.salone@ingv.it](mailto:rosanna.salone@ingv.it))

**Contents**

S1. Sensitivity to clay cap permeability

S2. Role of the main fault system

S3. Concluding remarks on sensitivity tests

Tables S1–S2

This file provides additional information to complement the main article. It includes details of sensitivity tests on cap/fault permeability and deep injection, as well as additional tables reporting input parameters and model outputs.

**S1. Sensitivity analysis on clay cap permeability**

The clay cap exerts a primary influence on shallow circulation and degassing. To test its role, we assigned alternative cap permeabilities and, for each scenario, adjusted the H_2_O–CO_2_ injection at the source cells to remain consistent with the assumed permeability-flux relationship (i.e., decreasing k strengthens the sealing capacity and reduces surface fluxes, whereas increasing k facilitates bypass and enhances surface fluxes). This experimental design enabled us to evaluate how the coupled response of injected flux, sub-cap pressure, and surface emissions changes with cap permeability.

The tests indicate that realistic Pisciarelli CO_2_ fluxes cannot be reproduced if the clay cap is treated as fully impermeable; a partially permeable cap is required instead. Since the imposed source flux varies with k, the simulated pressures do not change monotonically with permeability. For example, k = 1.0×10^-16^ m^2^ yields P ≈ 2.1 MPa, whereas k = 1.0×10^-17^ m^2^ yields P ≈ 1.1 MPa, reflecting the reduced injection rate required for the tighter-sealing cap (Fig. S1). Consequently, these results should be interpreted relative to each other, highlighting trends in sensitivity rather than providing absolute pressure estimates that can be compared directly with those of the calibrated model. Table S1 summarizes the permeability, imposed flux, pressure beneath the cap and maximum surface CO_2_ flux for each run.


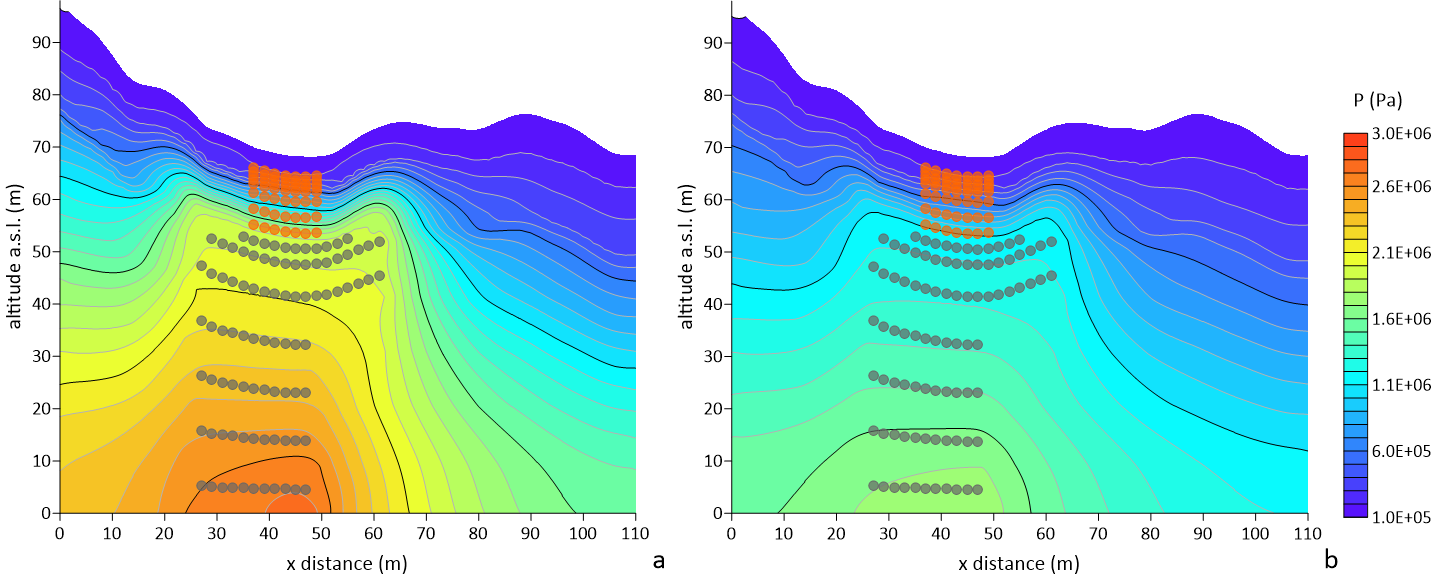


Figure S1. Simulated pressure profiles for scenarios T1 (a) and T2 (b) along section Y = 45 m, crossing the Soffione and the mud pool. The comparison shows how variations in the permeability of the clay cap affect the pressure distribution beneath the surface, with higher pressures being associated with the more permeable configuration (T1). Orange dots indicate the position of the clay cap, whereas grey dots mark the permeable channel.

**Table S1.**

*Results of the sensitivity tests on clay cap permeability. For each scenario (15,000 years), the permeability value, imposed H_2_O+CO_2_ flux, simulated pressure beneath the clay cap, and resulting CO_2_ flux at the surface are reported*

| **Test ID** | **Clay cap permeability (m^2^)** | **Imposed H_2_O+CO_2_** **flux (kg/s)** | **Simulated fluid pressure**  **(MPa)** | **Max surface CO_2_ flux**  **(g/m^2^day)** |
| --- | --- | --- | --- | --- |
| T1 | 1.00∙10^-16^ | 66.0 | 2.1 | 14.3 |
| T2 | 1.00∙10^-17^ | 26.4 | 1.1 | 3.57 |

**S2. Role of the main fault system**

To investigate the fault control, three configurations were explored (with cap permeability fixed at 5.0×10^-14^ m^2^ and a simulation time of 5,000 years): (i) fault absent; (ii) permeable fault present (k = 1.0×10^−12^ m^2^); and (iii) impermeable fault (k = 1.0×10^−17^ m^2^). In the impermeable configuration, a lower source flux was imposed to account for the sealing behaviour of the fault.

Fig. S2 shows the simulated fluid pressure distribution along section Y = 45 m, crossing the Soffione and mud pool, obtained at the final state of the numerical simulations for the three fault configurations.


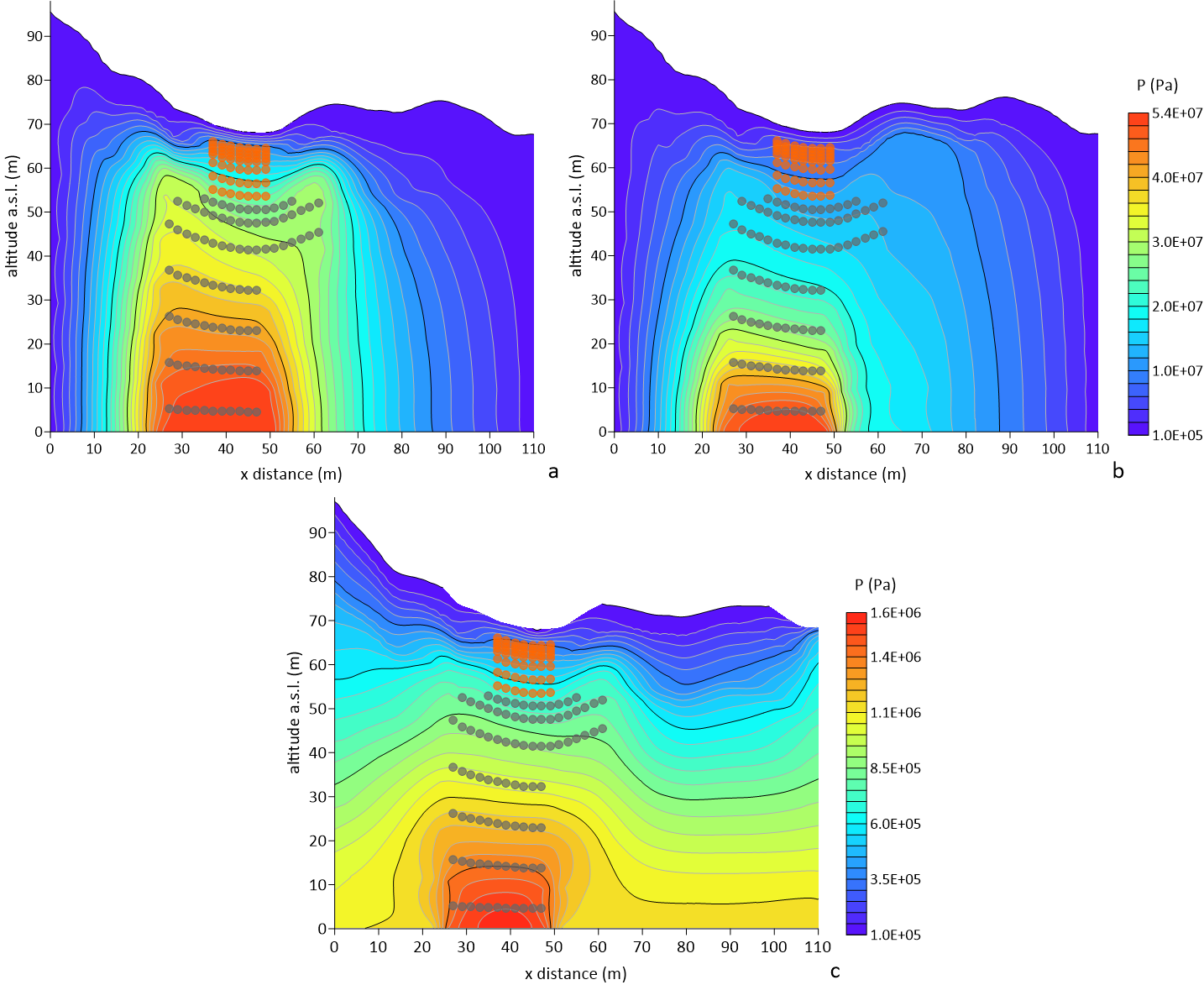


Figure S2. Simulated pressure profiles along section Y = 45 m, crossing the Soffione and the mud pool, for the three explored configurations: F1 (a) no fault, F2 (b) permeable fault, and F3 (c) impermeable fault. Orange dots indicate the position of the clay cap, while grey dots mark the permeable channel.

The outcomes show pronounced differences in pressure build-up beneath the clay cap and differ markedly in the magnitude of surface CO_2_ fluxes. In the absence of the fault, the system exhibits the highest pressures and fluxes (P ≈ 33.4 MPa; max CO_2_ flux of ~22,950 g/m^2^day). When the fault is permeable, the system is partly drained, resulting in reduced pressures and fluxes (P ≈ 12 MPa and a maximum CO_2_ flux of ~12,638 g/m^2^day). In the impermeable configuration, the simulated pressure beneath the cap is lower (P ≈ 0.8 MPa), resulting in a corresponding surface CO_2_ flux of ~4.50 g/m^2^day, because the imposed source flux is also lower in this scenario.

As the three scenarios employ different boundary conditions, particularly with regard to the imposed source flux, a direct comparison of absolute values is not meaningful. Instead, these simulations are intended to illustrate how variations in fault configuration redistribute fluid pathways and modulate the balance between focused and diffuse degassing. Table S2 summarizes the input parameters and resulting outputs.

**Table S2.**

*Results of the sensitivity tests on the role of the main fault. All simulations were run for 5,000 years with clay cap permeability fixed at 5.0×10^-14^* *m². Reported values include fault permeabilities, simulated fluid pressures beneath the clay cap, and maximum CO_2_ fluxes at the surface*

| **Test ID** | **Fault permeability (m^2^)** | **Simulated fluid pressure**  **(MPa)** | **Max surface CO_2_ flux**  **(g/m^2^day)** |
| --- | --- | --- | --- |
| F1 | - | 33.4 | 22,950 |
| F2 | 1.00∙10^-12^ | 12.0 | 12,638 |
| F3 | 1.00∙10^-17^ | 0.8 | 4.50 |

**S3. Conclusion remarks**

The sensitivity analyses emphasize the importance of structural permeability contrasts in determining plume geometry and degassing dynamics. Lower cap permeability reduces surface flux, favouring pressure build-up and lateral redistribution beneath the cap. In contrast, higher cap permeability facilitates drainage and enhances diffuse emissions. The fault acts as a system-scale valve: when it is permeable, it drains and weakens lateral pressure gradients, whereas when it is of low-permeability, it deflects upward flow and promotes accumulation along the channel-fault interface. Overall, these results support the interpretation given in the main text that the current state of the Pisciarelli hydrothermal system is influenced by the interaction between a partially permeable clay cap and a fault that primarily acts as a barrier. Together, these factors control fluid accumulation, lateral migration and the spatial and magnitude patterns of surface degassing.
